# Social network analysis by Turiyam graphs

**DOI:** 10.1186/s13104-023-06435-7

**Published:** 2023-08-14

**Authors:** Gamachu Adugna Ganati, V. N. Srinivasa Rao Repalle, Mamo Abebe Ashebo

**Affiliations:** Department of Mathematics, Wallaga University, Nekemte, Ethiopia

**Keywords:** Single valued neutrosophic graph, Turiyam graph, Degree, Order, Size, Social network, 05C07, 05C90, 05C99

## Abstract

**Objective:**

A single valued neutrosophic set represented the uncertainty of real life situations in terms of membership $(t)$, indeterminacy $(i)$ and non-membership $(f)$ degree. However, this uncertainty cannot be limited to those three degrees; there is also an additional refusal degree. For this issue, the Turiyam set is an appropriate tool, which described the neutrosophic refusal degree of this situation as a liberal $(l)$ degree in addition to those three degrees. The graphical representation of this situation is required for knowledge processing. For this purpose, the Turiyam graph was introduced as an extension of the single valued neutrosophic graph. This graph is helpful when the depictions of the vertices or their relationships or both, are considered in terms of membership $(t)$, indeterminacy $(i)$, non-membership $(f)$ and liberal $(l)$ degrees. The goal of this paper is to introduce the degree, order and size in the context of Turiyam graphs and examine a social network (SN) with the help of this graph.

**Results:**

In this regard, the degree, order and size in the context of Turiyam graphs are studied. The feasibility of this Turiyam graph is shown by employing its concept in a social network (SN). Finally, the advantage of the Turiyam graph over the existing graph theories is recognized by viewing its better framework.

## Introduction

Knowledge representation and processing is one of the major tasks for data science researchers. In this process, the data visualization can be considered as one of the prominent issues. To solve this issue, a mathematical model called graph theory was born as a humble mathematics subject by Euler in 1736 [1]. After Euler, many scholars like Kazimierz Kuratowski, Paul Erdös, and Dénes König were inclined the world of graph theory [2]. This subject has played a key role in solving relevant problems in different fields, like computer science, communications, sociology, chemistry, group theory and operations research. In classical graph theory, a vertex or edge is either part of the graph or not. Then, this graph cannot represent a situation when a vertex or edge or both are uncertain. Thus, we have different versions of graph, like a fuzzy graph (FG) [3], in which any vertex or edge is in the graph and an intuitionistic fuzzy graph (IFG) [4], in which there is uncertainty regarding whether any vertex or edge is in the graph or not, by adding non-membership values to the FG and a single valued neutrosophic graph (SVNG) (or simply neutrosophic graph) [5], in which there is indeterminacy regarding whether a vertex or edge is either part of the graph or not. The problem arises when we deal with the neutrosophic refusal degree (NRD) or $1-(t+i+f)$ of a vertex or edge in SVNG [6]*.* Consider the quality of a journal is not measured by its impact factor, indexing, or publisher but rather by its best authors and their publications [6]. In this case, a journal contains papers related to a given topic described as membership $\left(t\right),$ a journal does not contain a paper related to the given title described as non-membership$(f)$, the researcher is uncertain about the publication of his/her article in a journal described as indeterminate $\left(i\right)$ and the journal’s quality is good as it contains the researchers' papers related to the title of the article rather than indexing and impact factor represented as Turiyam $\left(l\right)$ [7]. It means selection of journal related to any topic is based on human Turiyam consciousness rather than indexed, not indexed or uncertain about indexing. In this case, the graph visualization of related or top researchers’ teams cannot be represented by SVNG. It is totally based on an expert Turiyam or liberal component that can be considered as fourth dimension consciousness. Recently, Turiyam sets [6, 7] were introduced as an extension of neutrosophic sets. The elements of this set are determined by the membership value $(t )$, the indeterminacy value $(i )$, the non-membership value $(f )$, and the liberal value $( l)$, all of which are in [0, 1]. The author applied this set to the voting system, sports data, medical diagnosis, identifying research paper quality, controlling car accidents and crime investigation systems [6, 7]. Also, Turiyam algebraic structures like Turiyam rings, Turiyam matrix, Turiyam spaces and Turiyam modules were studied [8–12]. This set is indeed a requirement to deal with the uncertainty in data sets beyond membership, non-membership and uncertainty, graphical visualization is required for dealing with these types of situations [13, 14]. Thus, Ganati et al $\left[15\right]$ developed a Turiyam graph based on Turiyam sets [6, 7] and Turiyam relations [16] to handle uncertainty with four dimensions where SVNG fails. The motive is to utilize the properties of Turiyam set in graph theory for its various applications like social networks [17, 18]. The authors defined some types of Turiyam graphs as a complete Turiyam graph, a strong Turiyam graph, and a constant Turiyam graph motivated by current studies [18–22]. To achieve this goal, some basic notation and its mathematical extension is required [16, 22]. Also, the authors applied the constant Turiyam graph to Wi-Fi technology. In the literature, this is the single study conducted on applications of the Turiyam graph. Thus, we are motivated to study other concepts to apply Turiyam graph in life situations like SN analysis. Numerous SN analyses are studied in the literature [17, 18, 23–25]. Accordingly, Koczy et al. [17] applied the concept of picture fuzzy graphs [PFGs] to SN analysis by handling abstinence and refusal degree of uncertainty where FG and IFG fail and showing that the sociable person in the given social network. Also, Akram et al. [18] applied the degree of single valued (di) graph to social network formed on whatsapp which contains indeterminate information and identified that the dominated and influenced persons in the given group. Many other precise uncertainties of network analysis are also handled by the concepts of graph theory [23–25]. There is a problem when we deal with the trustable person on the given social network. In this case, the trust values are based on human consciousness and it can be described either as trust on social network via action $(t)$, uncertainty trust on social network $(i)$, negative trust (or always opposing trust) on social network $(f)$ or hidden trust which is beyond any type of action shown on social network $(l)$. In such a situation, the Turiyam graph is appropriate to examine SN based on four membership degrees where FG, IFG and SVNG fail [16, 19–22]. In this way, the current paper defines the degree, order and size of the Turiyam graph and derives their properties. Further, we demonstrate the viability of this graph by utilizing its concept in a SN formed by individuals in the organization.

### Organization of the paper

This paper studies some properties like degree, order and size of Turiyam graphs. The next part of this work contains the review of the basic concepts, the description of the degree, order and size of Turiyam graphs, the proposed application of the concepts of Turiyam graphs in SN, the conclusion and limitation of the article.

Given $U$ is a universe set, $G=\left(V,E\right)$ is the classical graph theory and ${T}_{G}$ is a Turiyam graph of $G=\left(V,E\right)$.

#### Definition 1

[5] A SVNG on $U$ is a pair G = (N, R), where N is a single valued neutrosophic (SVN) set in $U$ and $R$ is (SVN) relation on $U$ such that

$${t}_{R}(ab)\le \mathrm{min}\{{t}_{N}\left(a\right),{t}_{N}\left(b\right)\}$$

$${i}_{R}(ab)\le \mathrm{min}\{{i}_{N}\left(a\right),{i}_{N}\left(b\right)\}$$

$${f}_{R}\left(ab\right)\le \mathrm{max}\left\{{f}_{N}\left(a\right),{f}_{N}\left(b\right)\right\},$$

$$\forall a,b\in U.$$

#### Definition 2

[6, 7] A Turiyam set $B$ on $U\ne \varnothing$ is a set of the form.

$$B =\left\{<x, {\mathrm{t}}_{B}\left(x\right), {i}_{B}\left(x\right), {f}_{B}\left(x\right),{l}_{B}\left(x\right)>:x\in U\right\}$$

where ${\mathrm{t}}_{B}\left(x\right),{i}_{B}\left(x\right),{ f}_{B}\left(x\right),{l}_{B}\left(x\right):U\to \left[\mathrm{0,1}\right]$ denote the membership value, the indeterminacy value, the falsity value and the liberal value, for each $x$ correspondingly by which ${\mathrm{t}}_{B}\left(x\right), {i}_{B}\left(x\right)$,${f}_{A}\left(x\right)$ and $l\left(x\right)$ satisfies

$${0\le \mathrm{t}}_{B}\left(x\right)+ {i}_{B}\left(x\right)+ {f}_{B}\left(x\right)+{l}_{B}\left(x\right)\le 4,\forall x\in U.$$

#### Definition 3

[16] Let A and B be two nonempty Turiyam sets on $U$.

- The Cartesian product of A and B,$A\times B$, is a Turiyam set in $U\times U$ defined as $A\times B=\left\{<\left(x,y\right), {t}_{A\times B}\left(x,y\right),{i}_{A\times B}\left(x,y\right),{f}_{A\times B}\left(x,y\right),{l}_{A\times B}\left(x,y\right)>:\left(x,y\right)\in A\times B\right\}$
  where ${t}_{A\times B},{i}_{A\times B},{f}_{A\times B},{l}_{A\times B}:U\to [\mathrm{0,1}]$ such that
  $${t}_{A\times B}\left(x,y\right)=\mathrm{min}\{{t}_{A}\left(x\right),{t}_{B}\left(y\right)\}$$
  $${i}_{A\times B}\left(x,y\right)=\mathrm{min}\{{i}_{A}\left(x\right),{i}_{B}\left(y\right)\}$$
  $${f}_{A\times B}\left(x,y\right)=\mathrm{max}\{{f}_{A}\left(x\right),{f}_{B}\left(y\right)\}$$
  $${l}_{A\times B}\left(x,y\right)=\mathrm{min}\{{l}_{A}\left(x\right), {l}_{B}\left(y\right)\}$$
- A relation from A to B is a Turiyam subset of $A\times B$ which has the form R = {${t}_{R}, {i}_{R},{f}_{R}, {l}_{R}\}$ where ${t}_{R}, {i}_{R},{f}_{R}, {l}_{R}: A\times B\to [\mathrm{0,1}]$ denote the truth membership, indeterminacy membership, falsity membership and liberation membership functions respectively.

#### Definition 4

[15] A Turiyam graph ${T}_{G}$ of $G=\left(V,E\right)$ on $U$ is an ordered pair ${T}_{G}=(A,R)$, where $A$ is the Turiyam vertex set and $R$ is the Turiyam edge set of ${T}_{G}$ such that.

$${t}_{R}\left({a}_{i}{b}_{j}\right)\le \mathrm{min}\left\{{t}_{A}\left({a}_{i}\right),{t}_{A}\left({b}_{j}\right)\right\}$$

$${i}_{R}\left({a}_{i}{b}_{j}\right)\le \mathrm{min}\left\{{i}_{A}\left({a}_{i}\right),{i}_{A}\left({b}_{j}\right)\right\}$$

$${f}_{R}\left({a}_{i}{b}_{j}\right)\le \mathrm{max}\left\{{f}_{A}\left({a}_{i}\right),{f}_{A}\left({b}_{j}\right)\right\}$$

$${l}_{R}\left({a}_{i}{b}_{j}\right)\le \mathrm{min}\begin{array}{c}\left\{{t}_{A}\left({a}_{i}\right),{t}_{A}\left({b}_{j}\right)\right\},\\ \forall {a}_{i},{b}_{j}\in V\end{array}$$

#### Example 1

**Fig. 1:**
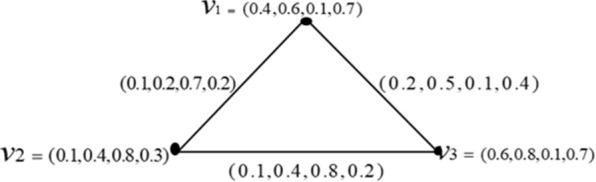
Turiyam graph

Consider a Turiyam graph $${T}_{G}=(A,R)$$ of $$G=\left(V,E\right)$$ where $$V=\{{v}_{1},{v}_{2},{v}_{3}\}$$ and $$E=\left\{{v}_{1}{v}_{2},{v}_{2}{v}_{3},{v}_{3}{v}_{1}\right\}. Fig.1 have to placed here$$

#### Definition 5

[15] A Turiyam graph ${T}_{H}=({A}{\prime},{R}{\prime})$ is a Turiyam subgraph of Turiyam graph ${T}_{G}=(A,R)$ if ${A}{\prime}\subseteq A$ and ${R}{\prime}\subseteq R.$

#### Definition 6

[15] A Turiyam graph ${T}_{G}=(A,R)$ of $G=\left(V,E\right)$ is a complete Turiyam graph if.

$${t}_{R}\left({a}_{i}{b}_{j}\right)=\mathrm{min}\left\{{t}_{A}\left({a}_{i}\right),{t}_{A}\left({b}_{j}\right)\right\}$$

$${i}_{R}\left({a}_{i}{b}_{j}\right)=\mathrm{min}\left\{{i}_{A}\left({a}_{i}\right),{i}_{A}\left({b}_{j}\right)\right\}$$

$${f}_{R}\left({a}_{i}{b}_{j}\right)=\mathrm{max}\left\{{f}_{A}\left({a}_{i}\right),{f}_{A}\left({b}_{j}\right)\right\}$$

$${l}_{R}\left({a}_{i}{b}_{j}\right)=\mathrm{min}\begin{array}{c}\left\{{t}_{A}\left({a}_{i}\right),{t}_{A}\left({b}_{j}\right)\right\},\\ \forall {a}_{i},{b}_{j}\in V\end{array}$$

### Degree, order and size in Turiyam graphs

#### Definition 7

Let ${T}_{G}$ be a Turiyam graph and $u$ be any vertex of a ${T}_{G}$. Then, the degree,$d\left(u\right),$ and the total degree, $Td\left(u\right),$ of u are given as.

$$d\left(u\right)=\left(\sum_{v\ne u}{t}_{R}\left(uv\right),\sum_{v\ne u}{i}_{R}\left(uv\right),\sum_{v\ne u}{f}_{R}\left(uv\right),\sum_{v\ne u}{l}_{R}\left(uv\right)\right)$$ and

$Td\left(u\right)=\left(\sum_{v\ne u}{t}_{R}\left(uv\right)+{t}_{A}\left(u\right),\sum_{v\ne u}{i}_{R}\left(uv\right)+{i}_{A}\left(u\right),\sum_{v\ne u}{f}_{R}\left(uv\right)+{f}_{A}\left(u\right),\sum_{v\ne u}{l}_{R}\left(uv\right)+{l}_{A}(u)\right)$ respectively, for $u \in V$ and $uv\in E$.

#### Example 2

Consider a Turiyam graph of Fig. 1. Then,

$$d\left({v}_{1}\right)=(\mathrm{0.3,0.7,0.8,0.6})$$ and

$Td({v}_{1})=\left(\mathrm{0.7,1.3,0.9,1.3}\right).$ It is similar for ${v}_{2}$ and ${v}_{3}.$

#### Theorem 1

*The sum of the degree of membership value, indeterminacy value, non-membership value and liberal value of all vertices in a* ${T}_{G}$ *is equal to twice the sum of the membership value, indeterminacy value, non-membership value and liberal value of all edges in* ${T}_{G}$ *respectively*.

#### Proof

Let ${T}_{G}=(A,R)$ be Turiyam graph such that $A=({u}_{1},{u}_{2},{\dots ,u}_{n})$. Then,

$$\begin{aligned} \sum d\left( {u_{i} } \right) =\, & [\sum d_{t} (u_{i} ),\sum d_{i} (u_{i} ),\sum d_{f} (u_{i} ),\sum d_{l} (u_{i} ),i = 1,2, \ldots ,n] \\ =\, & (d_{t} \left( {u_{1} } \right) + d_{i} \left( {u_{1} } \right) + d_{f} \left( {u_{1} } \right) + d_{l} \left( {u_{1} } \right)) + \left( {(d_{t} \left( {u_{2} } \right) + d_{i} \left( {u_{2} } \right) + d_{f} \left( {u_{2} } \right) + d_{l} \left( {u_{2} } \right)} \right) + \ldots + \left( {(d_{t} \left( {u_{n} } \right) + d_{i} \left( {u_{n} } \right) + d_{f} \left( {u_{n} } \right) + d_{l} \left( {u_{n} } \right)} \right) \\ \end{aligned}$$

$$=\,\left[\left(t\left({u}_{1}{u}_{2}\right),i\left({u}_{1}{u}_{2}\right),f\left({u}_{1}{u}_{2}\right),l\left({u}_{1}{u}_{2}\right)\right)+\left(t\left({u}_{1}{u}_{3}\right),i\left({u}_{1}{u}_{3}\right),f\left({u}_{1}{u}_{3}\right),l\left({u}_{1}{u}_{3}\right)\right)+\dots +\left(t\left({u}_{1}{u}_{n}\right),i\left({u}_{1}{u}_{n}\right),f\left({u}_{1}{u}_{n}\right),l\left({u}_{1}{u}_{n}\right)\right)+\left(t\left({u}_{2}{u}_{1}\right),i\left({u}_{2}{u}_{1}\right),f\left({u}_{2}{u}_{1}\right),l\left({u}_{2}{u}_{1}\right)\right)+\dots +\left(t\left({u}_{2}{u}_{n}\right),i\left({u}_{2}{u}_{n}\right),f\left({u}_{2}{u}_{n}\right),l\left({u}_{2}{u}_{n}\right)\right)+\dots +\left(t\left({u}_{n}{u}_{1}\right),i\left({u}_{n}{u}_{1}\right),f\left({u}_{n}{u}_{1}\right),l\left({u}_{n}{u}_{1}\right)\right)+\dots +\left(t\left({u}_{n-1}{u}_{n}\right),i\left({u}_{n-1}{u}_{n}\right),f\left({u}_{n-1}{u}_{n}\right),l\left({u}_{n-1}{u}_{n}\right)\right)\right],$$

$$=\,2[\left(t\left({u}_{1}{u}_{2}\right),i\left({u}_{1}{u}_{2}\right),f\left({u}_{1}{u}_{2}\right),l\left({u}_{1}{u}_{2}\right)\right)+\left(t\left({u}_{1}{u}_{3}\right),i\left({u}_{1}{u}_{3}\right),f\left({u}_{1}{u}_{3}\right),l\left({u}_{1}{u}_{3}\right)\right)+\dots +\left(t\left({u}_{1}{u}_{n}\right),i\left({u}_{1}{u}_{n}\right),f\left({u}_{1}{u}_{n}\right),l\left({u}_{1}{u}_{n}\right)\right)],\forall i=\mathrm{1,2},\dots ,n.$$

$$=[2\sum_{v\ne u}t\left(uv\right), 2\sum_{v\ne u}i\left(uv\right),2\sum_{v\ne u}f\left(uv\right),2\sum_{v\ne u}l\left(uv\right)] ,$$

Hence, the proof. □

#### Theorem 2

*The maximum degree of any vertex in a* ${T}_{G}$ *with* $n$ *number of vertices is* $n-1$.

#### Proof

Let ${T}_{G}=(A,R)$ be a TG with n number of vertices and $u$ be any vertex of ${T}_{G}.$ The membership value given to an edge is at most $1$ and the maximum number of edges incident on u can be $n-1$. Then, the degree of membership value of $u$ is $n-1$. Similarly, the degree of indeterminacy value, the degree of non-membership and the degree of liberal value of $u$ is $n-1$. Then, the maximum degree of $u$ is $n-1$. Hence, the proof. □

#### Definition 8

An edge $$e=uv$$ of a Turiyam graph $${T}_{G}$$ is an effective edge if the $$tv,iv,fv and lv$$ given by $$t\left(uv\right)=t\left(u\right)\bigwedge t\left(v\right),i\left(uv\right)=i\left(u\right)\bigwedge i\left(v\right),f\left(uv\right)=f\left(u\right)\bigvee f\left(v\right)$$ and $$l\left(uv\right)=l\left(u\right)\bigwedge l\left(v\right),\forall e\in R$$ respectively. In this case, $$u$$ is a neighborhood of $$v$$ and vice versa.

#### Remark

$N(u)=\{v\in A: v$ is a neighborhood of $u\}$ is a neighborhood of $u$.

#### Example 3

**Fig. 2:**
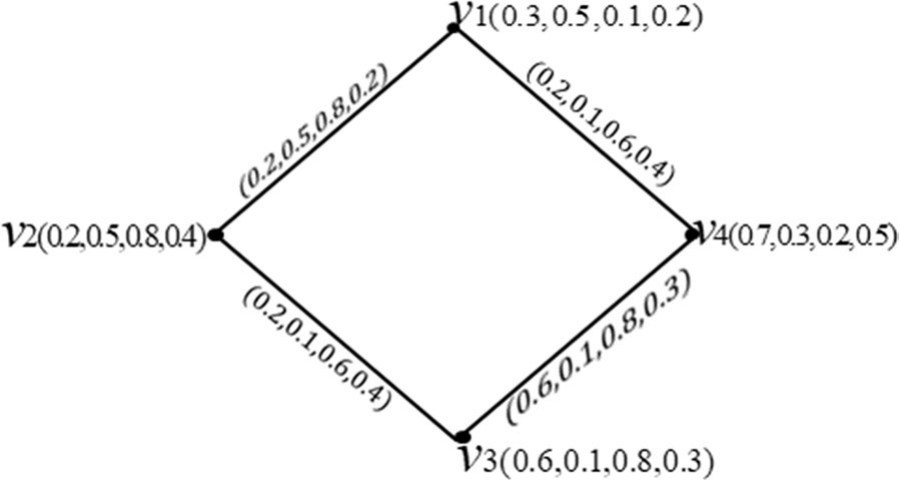
Turiyam graph

Consider a Turiyam graph ${T}_{G}=\left(A,R\right)$ such that $A=\left\{{v}_{1},{v}_{2},{v}_{3},{v}_{4}\right\}and$

$$R=\left\{{v}_{1}{v}_{2},{v}_{2}{v}_{3},{v}_{3}{v}_{4},{v}_{4}{v}_{1}\right\}. Fig.2 have to placed here$$

Now, ${v}_{1}{v}_{2}$ and ${v}_{3}{v}_{4}$ are effective edges. Then, $N\left({v}_{4}\right)=\left\{{v}_{1},{v}_{3}\right\}$ is a neighborhood of ${v}_{4}$.

#### Definition 9

The effective degree of a vertex $u$ ${,d}_{e}\left(u\right),$ in ${T}_{G}$ is defined by ${d}_{e}\left(u\right)=({d}_{et}\left(u\right),{d}_{ei}\left(u\right),{d}_{ef}\left(u\right),{d}_{el}\left(u\right))$ where ${d}_{et}\left(u\right)$ is the sum of the membership values of effective edges, ${d}_{ei}\left(u\right)$ is the sum of the indeterminacy values of effective edges, ${d}_{ef}\left(u\right)$ is the sum of the non-membership values of effective edges and ${d}_{el}\left(u\right)$ is the sum of the liberal values of effective edges incident to $u$.

#### Definition 10

The minimum effective degree $({m}_{ed})$ of ${T}_{G}$ is ${m}_{ed}\left({T}_{G}\right)=({m}_{et}\left(G\right),{m}_{ei}\left(G\right),{m}_{ef}\left(G\right),{m}_{el}\left(G\right))$ where ${m}_{et}\left(G\right)$ represents the ${m}_{ed}$ of $t$,${m}_{ei}\left(G\right)$ represents the ${m}_{ed}$ of $i$, ${m}_{ef}\left(G\right)$ represents the ${m}_{ed}$ of $f$ and ${m}_{el}\left(G\right)$ represents the ${m}_{ed}$ of $l$. Similarly, we define the maximum effective degree ${(M}_{ed})$ of ${T}_{G}.$

#### Example 4

Consider the Turiyam graph of Fig. 2. Then,

$${d}_{e}\left({v}_{1}\right)=\left(\mathrm{0.2,0.5,0.8,0.2}\right)={d}_{e}\left({v}_{2}\right),{d}_{e}\left({v}_{3}\right)=\left(\mathrm{0.6,0.1,0.8,0.3}\right)={d}_{e}\left({v}_{4}\right)$$

$$, {m}_{ed}\left({T}_{G}\right)=\left(\mathrm{0.2,0.1,0.8,0.2}\right)and {M}_{ed}\left({T}_{G}\right)=\left(\mathrm{0.6,0.5,0.8,0.3}\right).$$

#### Definition 11

Let ${T}_{G}=(A,R)$ be a Turiyam graph. The neighborhood of a vertex $u$ of ${T}_{G}$ is given by $N(u)=\left({n}_{t}\left(u\right),{n}_{i}\left(u\right),{n}_{f}\left(u\right),{n}_{l}\left(u\right)\right)$ where ${n}_{t}\left(u\right)=\{t\left(uv\right)=t\left(u\right)\wedge t\left(v\right);v\in A\}$ represents the neighborhood t-vertex, ${n}_{i}\left(u\right)=\{i\left(uv\right)=i\left(u\right)\wedge i\left(v\right);v\in A\}$ represents the neighborhood $i$-vertex,${n}_{f}\left(u\right)=\{f\left(uv\right)=f\left(u\right)\vee f\left(v\right);v\in A\}$ represents the neighborhood $f$-vertex and ${n}_{l}\left(u\right)=\{l\left(uv\right)=l\left(u\right)\wedge l\left(v\right);v\in A\}$ represents the neighborhood $l$-vertex.

In this case, $[N]= N\left(u\right)\cup \{u\}$ is said to be the closed neighborhood of $u$.

#### Definition 12

Let ${T}_{G}=(A,R)$ be a Turiyam graph. Then the open neighborhood degree (OND) of a vertex $u$ of ${T}_{G}$ is given as the sum of membership, indeterminacy, falsity and liberal value of the neighborhood vertices of $u$, ${d}_{n}(u)$.

#### Definition 13

Let ${T}_{G}=(A,R)$ be a Turiyam graph. Then the minimum OND of ${T}_{G}$ is given as the minimum of membership, indeterminacy, falsity and liberal value of the neighborhood degree vertices of ${T}_{G}$, ${d}_{mn}({T}_{G})$. Similarly, we define the maximum OND of ${T}_{G}$,${d}_{Mn}\left({T}_{G}\right).$

#### Example 5

Consider the Turiyam graph of Fig. 1.Then,

$${d}_{n}\left({v}_{1}\right)=\left(0.7, 1.2, 0.9, 1\right),{d}_{n}\left({v}_{2}\right)=(1, 1.4, 0.2, 1.4),{d}_{n}\left({v}_{3}\right)=(0.5, 1, 0.9, 1)$$, $${d}_{mn}\left({T}_{G}\right)=(0.5, 1, 0.2, 1)$$ and $${d}_{Mn}\left({T}_{G}\right)=(1, 1.4, 0.9, 1.4)$$.

#### Definition 14

Let ${T}_{G}=(A,R)$ be a Turiyam graph. Then the closed neighborhood degree (CND) of a vertex $u$ of ${T}_{G}$ is given as the sum of ${d}_{n}(u)$ and $u$. We write ${d}_{n}[u]$.

#### Definition 15

The minimum CND of ${T}_{G},{m}_{cnd}\left({T}_{G}\right),$ is ${m}_{cnd}\left({T}_{G}\right)=({m}_{cndt}\left(G\right),{m}_{cndi}\left(G\right),{m}_{cndf}\left(G\right),{m}_{cndl}\left(G\right))$ where ${m}_{cndt}\left(G\right)$ represents the minimum CND of $t$,${m}_{cndi}\left(G\right)$ represents the minimum CND of $i$, ${m}_{cndf}\left(G\right)$ represents the minimum CND of $f$ and ${m}_{cndl}\left(G\right)$ represents minimum CND of $l$. Similarly, we define the maximum CND of ${T}_{G},{M}_{cnd}\left({T}_{G}\right).$

#### Example 6

The ONDs for all vertices, minimum and maximum CNDs of Turiyam graph given in Fig. 1 are ${d}_{n}\left[{v}_{1}\right]=\left(1.1, 1.8, 1, 1.7\right)={d}_{n}\left[{v}_{2}\right]={d}_{n}\left[{v}_{3}\right]$ and ${m}_{cnd}\left({T}_{G}\right)={M}_{cnd}\left({T}_{G}\right)=\left(1.1, 1.8, 1, 1.7\right).$

#### Definition 16

A Turiyam graph ${T}_{G}$ is a regular Turiyam graph if all the vertices have the same CNDs.

#### Theorem 3

*Every complete Turiyam graph is regular Turiyam graph*.

#### Proof

Let ${T}_{G}=(A,R)$ be a complete Turiyam graph. Then, $t(uv)= t(u)\bigwedge t(v), i(uv)= i(u)\bigwedge i(v), f(uv)= f(u)\bigvee f(v) and l(uv)= l(u)\bigwedge l(v),\forall u,v\in A.$

By definition, the membership CND of each vertex is the sum of the membership values of the vertices and itself. It is similar for the indeterminacy CND, the non-membership CND and the liberal CND of each vertex of ${T}_{G}$. Then, all the vertices will have the identical neighborhood degree. This implies the minimum CND of ${T}_{G}$ is equal with the maximum CND of ${T}_{G}$. Hence, the proof. □

#### Definition 17

Let ${T}_{G}=(A,R)$ be a Turiyam graph.

- The order of ${T}_{G}$ ${,O(T}_{G}),$ is defined as the number of vertices.
- The size of ${T}_{G}$ ${,S(T}_{G}),$ is defined as the number of edges.

#### Example 7

Consider the Turiyam graph of Fig. 2. Then,${O(T}_{G})=(1.8, 1.4, 1.9, 1.4)$ and ${S(T}_{G})=\left(1.2, 0.8, 2.8, 1.3\right).$

#### Theorem 4

*In a complete Turiyam graph* ${T}_{G}$*, the CND of any vertex of *${T}_{G}$ *is the same with the order of neighborhood *degrees of ${T}_{G}$.

#### Proof

Let ${T}_{G}=(A,R)$ be a complete TG. Then, the $t$-order of ${T}_{G}$ is the sum of the membership values of all vertices and similarly the $i$-order, the $f$-order and the $l$-order of ${T}_{G}$ can be obtained. Since ${T}_{G}$ is complete TG, the $t$- CND of any vertex is the sum of the membership value of vertices and it is similar for the $i$- CND, $f$- CND, $l$- CND of any vertex of ${T}_{G}$. Hence, the proof. □

### Application to social network

We use graph theory to handle our daily life. One of its crucial roles is to examine SN problems. A SN consists of a set of existing social bodies and sets of links among them. One of the important functions of SNs is to measure trust among the workers of the organization. In this section, we describe the uncertainty in SNs based on four membership degrees where FG, IFG and SVNG fail. Thus, we apply a ${T}_{G}$ to handle this situation.

Consider a set $$D=\{Gadisa, Hasan, Hana, Keneni, Fikadu,Birraa,Michu,$$

$Badhasa, Lamessa,Tame, Soressa\}$ of eleven individuals in social group of certain organization.

Let $\mathrm{A}=\{<\mathrm{Gadisa}, (0.2, 0.1, 0.3, 0.5) >,<\mathrm{ Hasan}, (0.5, 0.3, 0.2, 0.5) >, <\mathrm{Hana},(\mathrm{0.6,0.4}, 0.9, 0.8)>,<\mathrm{ Keneni}, (0.4, 0.5,\mathrm{ 0.3,0.7})>, <\mathrm{Fikadu},(\mathrm{0.8,0.7}, 0.3, 0.6)>, <\mathrm{Birraa},(\mathrm{0.4,0.6,0.5,0.3})>,<\mathrm{Michu},(\mathrm{0.7,0.4},\mathrm{ 0.6,0.5})>, <\mathrm{Badhasa},(\mathrm{0.3,0.4,0.1,0.6})>, <\mathrm{Lamessa},(0.2, 0.4, 0.5, 0.4)>, <\mathrm{Tame}, (1, 0.2, 0.3, 0.4)>, <\mathrm{Soressa},(0.5,\mathrm{ 0.2,0.6,0.6})>\}$ be the Turiyam set on the set $D.$

Let $$E = \{ (Hassan , Lamessa), (Soressa , Keneni), (Lamessa, Soressa), (Fikadu, Lamessa),$$

$$\left(Hana, Keneni\right), \left(Birraa, Michu\right),$$

$$\left(Gadisa, Hassan\right),$$

$$\left(Birraa, Fikadu\right), \left(Hassan, Tame\right),$$

$(Fikadu, Hassan), (Badhasa, Keneni)\}$ be the set of edges.

Consider the Turiyam set $B$ defined on the set E shown by Table 1.

**Table 1:** Edge values for SN of Fig. 3

Relation	Edges	Values
Hassan–Lamessa	${e}_{1}$	(1, 0.3, 0.4, 0.2)
Soressa–Keneni	${e}_{2}$	(0.4, 0.2, 0.3, 0.5)
Lamessa–Soressa	${e}_{3}$	(0.2, 0.1, 0.5, 0.3)
Fikadu–Lamessa	${e}_{4}$	(0.0, 0.4, 0.1, 0.2)
Hana–Keneni	${e}_{5}$	(0.1, 0.3, 0.5, 0.4)
Birraa–Michu	${e}_{6}$	(0.4, 0.3, 0.5, 0.2)
Gadisa–Hassan	${e}_{7}$	(0.0, 0.1, 0.1, 0.3)
Birraa–Fikadu	${e}_{8}$	(0.3, 0.0, 0.4, 0.2)
Hassan–Tame	${e}_{9}$	(0.5, 0.1, 0.0, 0.0)
Fikadu–Hassan	${e}_{10}$	(0.3, 0.2, 0.1, 0.5)
Badhasa–Keneni	${e}_{11}$	(0.1, 0.3, 0.3, 0.0)

**Fig. 3:**
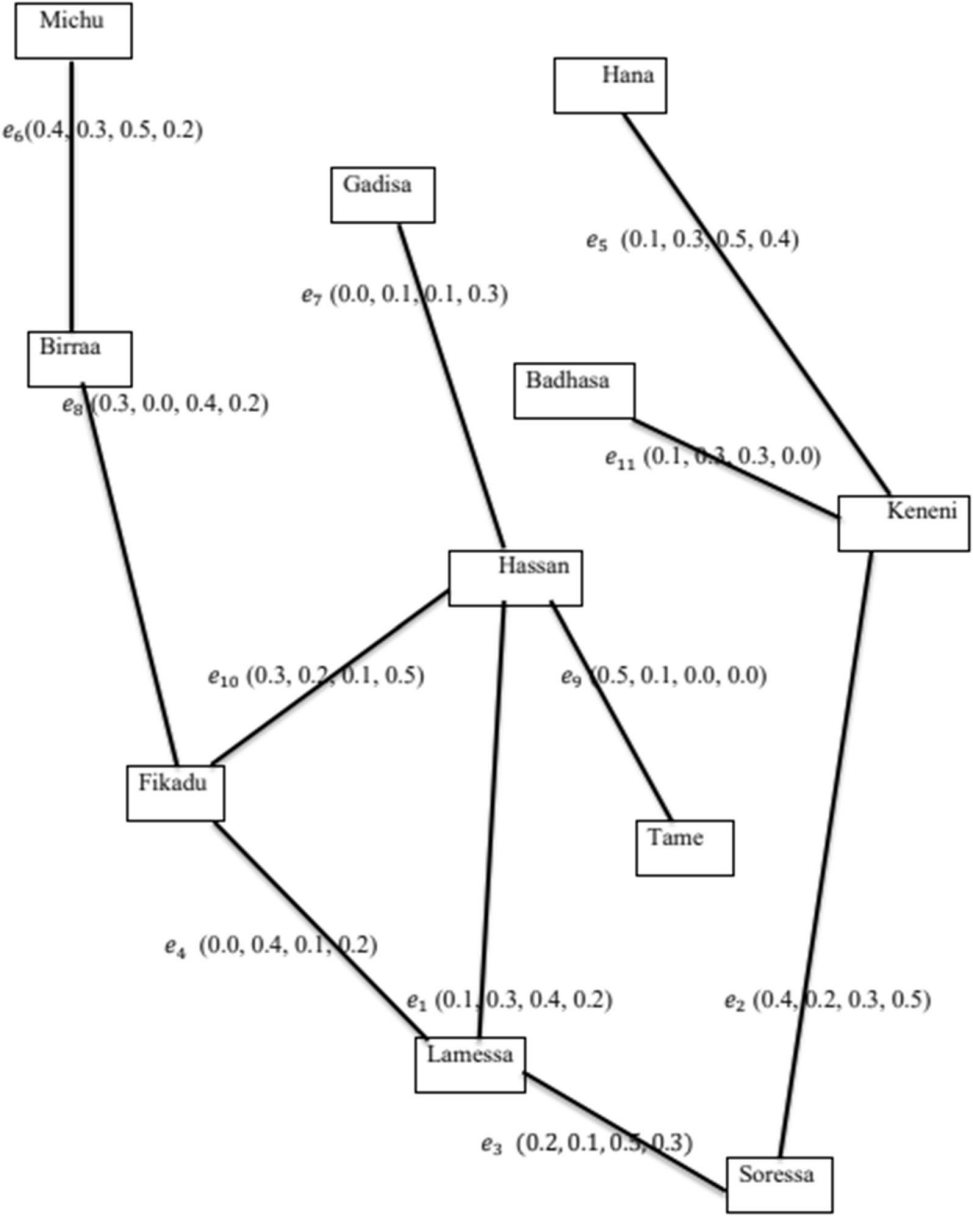
Social network of set of staff

In those edges, the 1st component $(t)$ shows true trust showing trust or supports on SN via action; the 2nd component $\left(i\right)$ shows uncertain trust as showing uncertainty act on SN, the 3^rd^ component $(f)$ shows false trust showing negative trust or always opposing on SN and the 4^th^ component $(l)$ shows hidden trust which is beyond any type of action shown on SN. This Turiyam trust relation is beyond the true, false and uncertain relation. It means two people may have closed friendship beyond the social network which known to only two person. It is a Turiyam relation or liberal relationship [16]. It means the larger the Turiyam (or liberal) value of a person is, the more trustworthy and vice versa. Thus, the person knows he/she is may a true friend beyond the action of SN.

In this table, larger liberal value shows more trustable values of a person and vice versa. The SN connecting diverse individuals is given in the figure below.

To know the trustable person beyond the action of SN in the given SN, first we compute the degree of each vertex in the given Turiyam graph as shown in Table 2.

**Table 2:** Degree of nodes for SN of Fig. 3

Nodes	Degree of node
Hassan	(0.9, 0.7, 0.6, 1)
Soressa	(0.6, 0.3, 0.8, 0.8)
Lamessa	(0.3, 0.8, 1, 0.7)
Fikadu	(0.6, 0.6, 0.6, 0.9)
Hana	(0.1, 0.3, 0.5, 0.4)
Birraa	(0.7, 0.3, 0.9, 0.4)
Gadisa	(0.0, 0.1, 0.1, 0.3)
Tame	(0.5, 0.1, 0.0, 0q.0)
Michu	(0.4, 0.3, 0.5, 0.2)
Badhasa	(0.1, 0.3, 0.3, 0.0)
Keneni	(0.6, 0.8, 1.1, 0.9)

In the above table, the more the degree of an individual means, the more trustable individual.

Now, to identify the most trustable individual, we calculate the NRD of the degree in Table 2.

In Table 3, the NRD value of Lamessa is 2.5, Keneni is 2.3, Birraa is 1.7, Fikadu is 1.6, three people (Soressa, Birraa and Badhasa) is 1.5, two people (Hassan and Michu) is 1.4, Gadisa is 1.2 and Tame is 0.6. Then Lamessa is the most trustable person in the given SN. Accordingly, we ranked the rest as 2nd, 3rd, 4th, 5th, 6th, 7th and 8th based on their values in this SN. In this way, the Turiyam set and its graphical representation provides an alternative way to deal with SN data sets [19–22].

**Table 3:** The NRD of the degree calculated in Table 2

Nodes	NRD of degree
Hassan	1.4
Soressa	1.5
Lamessa	2.5
Fikadu	1.6
Hana	1.7
Birraa	1.5
Gadisa	1.2
Tame	0.6
Michu	1.4
Badhasa	1.5
Keneni	2.3

We give the procedure of the given application by the algorithm as follows:

Step 1. Input the set of vertices.

$D=\left\{{d}_{1},{d}_{2},\dots ,{d}_{n}\right\}$ and a Turiyam set A which given on set.

Step 2. Input the set of edges

$$E=\left\{{e}_{1},{e}_{2},\dots ,{e}_{n}\right\}.$$

Step 3. Find the membership degree (t), indeterminacy degree (i), non-membership degree (f) and liberal degree (l) of every edge as ${ t}_{R}\left(ab\right)\le \mathrm{min}\left\{{t}_{A}\left(a\right),{t}_{A}\left(b\right)\right\},$ ${i}_{R}\left(ab\right)\le \mathrm{min}\left\{{i}_{A}\left(a\right),{i}_{A}\left(b\right)\right\}$, ${f}_{R}\left(ab\right)\le \mathrm{max}\left\{{f}_{A}\left(a\right),{f}_{A}\left(b\right)\right\}$ and ${l}_{R}\left(ab\right)\le \mathrm{min}\left\{{l}_{A}\left(a\right),{l}_{A}\left(b\right)\right\}$.

Step 4. Compute the Turiyam set B of edges.

Step 5. Give a Turiyam graph ${T}_{G}=(A,R)$.

### Comparative analysis

Both PFGs [17] and SVNGs [18] model the uncertainty by using three degrees for each vertex where FG and IFG fail. Ganati et.al $\left[15\right]$ have described the application of the Turiyam graph on wireless network. In this work, four degrees of vertex are required to describe the trusted person on SNs. That is, trust value can be expressed on social network either via action, uncertainty, negative (oppose) or beyond any type of action. Therefore, the graphs like FG, IFG, PFG and SVNG fail to handle this situation. However, the degree of vertices in the Turiyam graph is the appropriate way to describe this situation. Here, first we constructed the Turiyam graph of a given SN of a certain organization. Then, we find the degree of each vertex in SN and finally we compute the NRD of the calculated degree. Based on the highest NRD, the most trustable person on SN is identified.

## Conclusion

In this paper, some concepts of the Turiyam graph like degree, order and size are described and some of their properties were derived. The degree of vertices in the Turiyam graph is used to examine the SN analysis of certain individuals of the organization. The novelty of the Turiyam graph over other graphs like SNG, IFG and FG, due to its fourth degree, is shown by providing an alternative way to deal with SN. In the near time, we will extend the concept to crime pattern analysis and, based on this characteristic of vertices and edges of Turiyam graphs, we extend the concept to strong degrees in Turiyam graphs.

## Limitations

- This paper is limited to the study of the degree, order and size of Turiyam graphs and its applications in SNs.
- Turiyam graph is appropriate for uncertainty of SN with four degrees.
- The concept of Turiyam graph is applicable only for Turiyam environment.

## Data Availability

Not applicable.
